# Phenanthrene and Pyrene Modify the Composition and Structure of the Cultivable Endophytic Bacterial Community in Ryegrass (*Lolium multiflorum* Lam)

**DOI:** 10.3390/ijerph13111081

**Published:** 2016-11-03

**Authors:** Xuezhu Zhu, Li Jin, Kai Sun, Shuang Li, Xuelin Li, Wanting Ling

**Affiliations:** Institute of Organic Contaminant Control and Soil Remediation, College of Resource and Environmental Sciences, Nanjing Agricultural University, Nanjing 210095, China; zhuxuezhu@njau.edu.cn (X.Z.); jinlinj0411@126.com (L.J.); 2013203015@njau.edu.cn (K.S.); 2015103062@njau.edu.cn (S.L.); lixuel@njau.edu.cn (X.L.)

**Keywords:** community structure, cultivable endophytic bacteria, diversity, biodegradation, polycyclic aromatic hydrocarbons

## Abstract

This study provides new insights into the dynamics of bacterial community structure during phytoremediation. The communities of cultivable autochthonous endophytic bacteria in ryegrass exposed to polycyclic aromatic hydrocarbons (PAHs) were investigated with regard to their potential to biodegrade PAHs. Bacterial counts and 16S rRNA gene sequence were used in the microbiological evaluation. A total of 33 endophytic bacterial strains were isolated from ryegrass plants, which represented 15 different genera and eight different classes, respectively. Moreover, PAH contamination modified the composition and structure of the endophytic bacterial community in the plants. *Bacillus* sp., *Pantoea* sp., *Pseudomonas* sp., *Arthrobacter* sp., *Pedobacter* sp. and *Delftia* sp. were only isolated from the seedlings exposed to PAHs. Furthermore, the dominant genera in roots shifted from *Enterobacter* sp. to *Serratia* sp., *Bacillus* sp., *Pantoea* sp., and *Stenotrophomonas* sp., which could highly biodegrade phenanthrene (PHE). However, the diversity of endophytic bacterial community was decreased by exposure to the mixture of PAHs, and increased by respective exposure to PHE and pyrene (PYR), while the abundance was increased by PAH exposure. The results clearly indicated that the exposure of plants to PAHs would be beneficial for improving the effectiveness of phytoremediation of PAHs.

## 1. Introduction

Polycyclic aromatic hydrocarbons (PAHs), which are potentially toxic, mutagenic, and carcinogenic, are widespread in Nature because of polluting anthropogenic activities [1,2]. Soil contaminated with PAHs may exhibit toxicity towards different plants, microorganisms and invertebrates. Among these PAHs, phenanthrene (PHE) is listed as a priority pollutant by the U.S. Environmental Protection Agency, and pyrene (PYR) has been widely used as an indicator and model compound to study biodegradation of high-molecular-weight (HMW) PAHs [3]. Therefore, in this study PHE and PYR were selected to be representative PAHs.

Phytoremediation is the most promising and environmentally friendly method that has been proposed for PAH degradation, because of its low cost and positive impact on the soil. In phytoremediation, plants are known to enhance the remediation of soil via biophysical and biochemical processes, such as adsorption of nutrients bound with pollutants, uptake of pollutants, secretion of enzymes, capability to store pollutants, and chemical transformation of toxic elements into relatively harmless forms [4]. On the other hand, the presence and activity of plant-associated microorganisms are vital influences on the effectiveness of phytoremediation. Endophytic bacteria may stimulate plant growth, suppress pathogens, and help to remove pollutants from plant tissues. Ryegrass (*Lolium multiflorum* Lam), whose fibrous root system provides a large surface area for plant-associated microbial colonization, has been widely used in the cleanup of PAH-contaminated soils [5]. However, a better understanding of ryegrass-associated microorganisms is beneficial for improving the effectiveness of phytoremediation.

It has been demonstrated previously that synergistic interactions between plants and microbial communities colonizing inside plant tissues may contribute to the degradation of recalcitrant organic compounds [6,7,8]. The presence of PAHs potentially stimulates the degradative potential of endophytic bacteria by changing the composition and structure of their community [9]; however, the particular mechanism of this occurrence is still unclear.

Studies on endophytic bacteria are important not only for understanding their ecological role in interactions with plants, but also for their possible biotechnological applications in phytoremediation. In this experiment, ryegrass was chosen to be the host plant. Since the most significant genera in the total communities are indeed cultivable under laboratory conditions [9], the cultivable communities were identified, and the microbial communities related to PAH degradation were analyzed. The objectives of this study were: (1) to assess the structure of endophytic bacterial communities in ryegrass exposed to PAHs; (2) to determine the degradation potential of endophytic isolates; (3) and to isolate highly PAH-degrading endophytic bacteria. This study provides new insights into the dynamics of endophytic bacterial community structure during phytoremediation process and a possible way to enhance the remediation of soil.

## 2. Materials and Methods

### 2.1. Experimental Materials

Ryegrass (*Lolium multiflorum* Lam) used as the host plant in this study was purchased from the Jiangsu Academy of Agricultural Science (Nanjing, China). PHE and PYR (purity > 98% by high-performance liquid chromatography (HPLC)) purchased from Fluka Chemical Co. (Milwaukee, WI, USA), were selected as representative PAHs. All solvents and chemicals used in this study were analytical grade reagents.

### 2.2. Exposure of Plants to PAHs

Six treatments (T_1_, T_2_, T_3_, T_4_, T_5_ and T_6_) and one control (CK) were performed in this test. The seedlings from these groups were grown in the Hoagland solution with different concentrations of phenanthrene and pyrene (Table 1). Surface-sterilized seeds of ryegrass were germinated and cultivated in 1/5 Hoagland solution for 48 h until 10-cm length, then exposed to PAHs for 7 d. Seedlings were transplanted into 300-mL brown color flasks sealed with translucent caps. Each flask contained 250 mL Hoagland solution for 10 seedlings. Plants were kept in a growth chamber with a 12 h photoperiod and a light: dark temperature regime of 25:20 °C. The relative humidity was 50%–60%. For the isolation of endophytic bacteria, flasks with seedlings were sampled on the 7th day in triplicate.

### 2.3. Isolation and Identification of Endophytic Bacteria

Prior to isolation of endophytic bacteria from *Lolium multiflorum* seedlings, plant tissues were sterilized by immersion in 75% (*v*/*v*) ethanol-water solution for 3–5 min, followed by immersion in 0.1% (*v*/*v*) mercuric chloride solution for 2–5 min. Subsequently, plant tissues were washed with sterile deionized water three times to remove the surface sterilization agents, and then plant tissues were cultivated on a Luria-Bertani (LB) plate for confirmation that all external bacteria were eliminated. After being successfully surface-disinfected, the ryegrass tissues were ground aseptically. The extract was incubated on LB medium agar plates at 30 °C for 72 h. Selection of bacterial strains was based on the morphology and colour of isolated colonies. Classification of bacterial strains was based on 16S rRNA gene sequence analysis. Amplification of 16S rRNA gene fragment was performed according to the method described by Hung and Annapurna [10], using the universal primers, 16S-27F and 16S-1492R (Invitrogen Co. Ltd., Shanghai, China). The 25-μL polymerase chain reaction (PCR) mixture contained 1-μL template, 2.5 μL of 10× Taq DNA polymerase buffer, 5 mmol·L^−1^ MgCl_2_, 1-μL dNTPs at 2.5 mmol·L^−1^, 3.75 pmol each of the forward and reverse primers, and 0.5 mL of 2.5 units Taq polymerase. The sequencing was performed at Nanjing Genscript Biotechnology Company, Limited (Nanjing, China). The 16S rRNA gene sequences were queried against the GenBank database [11] and the microgenetic analysis was performed using MEGA 6.0 program.

For analysis of the diversity of endophytic bacterial community, the Shannon-Wiener index (*H*) and Evenness (*J*) were calculated using Equations (1) and (2), respectively:

(1) H=∑pi ln pi

where *pi* is the proportion of species *i* in the seedlings from the same group;

(2) $$J=H/H_{\text{max}}$$

where *H*_max_ is the maximum value of *H* for the number of species (*S*) present (*H*_max_ = ln*S*) [12].

### 2.4. Degradation of PAHs by Endophytic Bacteria

The enrichment of cultures capable of degrading PHE and PYR was also studied for each strain of endophytic bacterium, which was a dominant in each treatment. Biodegradation of PAHs was monitored in 50-mL flasks containing 20 mL mineral salt medium (MS medium) with a final concentration of 50 mg·L^−1^ for PYR or 100 mg·L^−1^ for PHE as the sole carbon source. The MS medium contained 1.50 g·L^−1^ of (NH_4_)_2_SO_4_, 1.91 g·L^−1^ of K_2_HPO_4_·3H_2_O, 0.50 g·L^−1^ of KH_2_PO_4_, 0.20 g·L^−1^ of MgSO_4_·7H_2_O, and 1 mL of trace element solution (0.1 mg·L^−1^ of CoCl_2_·6H_2_O, 0.425 mg·L^−1^ of MnCl_2_·4H_2_O, 0.05 mg·L^−1^ of ZnCl_2_, 0.01 mg·L^−1^ of NiCl_2_·6H_2_O, 0.015 mg·L^−1^ of CuSO_4_·5H_2_O, 0.01 mg·L^−1^ of Na_2_MoO_4_·2H_2_O, and 0.01 mg·L^−1^ of Na_2_SeO_4_·2H_2_O). A 1-mL aliquot (10^8^ CFU·mL^−1^) of each strain suspension was added to 20 mL MSM with PAHs. Flasks were inoculated with sterilized MS medium to assess abiotic influences on the attenuation of PAHs. 7 d after the cultivation, three flasks from each treatment were taken and the residual PHE/PYR was detected.

### 2.5. Concentrations of PAHs

PHE/PYR was extracted from the media with methanol, which was added to the medium at the ratio 7:3 (*v*/*v*). After ultrasonic extraction for 30 min, samples were centrifuged at 12,000× *g* for 10 min and filtered through 0.22 µm filters.

PAHs in seedlings were extracted as follows: plant samples were freeze-dried, ground and homogenized. After ultrasonic extraction in 1:1 (*v*/*v*) acetone and dichloromethane (DCM) solution with anhydrous Na_2_SO_4_ for 30 min, the extract was centrifuged at 12,000× *g* for 10 min; the extraction procedure was repeated three times. All supernatants were loaded on a 2 g silica gel column (Bonna-Agela Technologies, Limited, Tianjin, China), which was eluted with 10 mL 1:1 (*v*/*v*) DCM and hexane. Samples were evaporated, then dissolved in methanol in a final volume of 10 mL and filtered through a 0.22-µm filter unit.

Concentrations of PAHs in prepared samples were quantified by HPLC (LC-20AT; Shimadzu, Kyoto, Japan) equipped with a 4.6 × 150-mm reverse-phase C_18_ column using methanol/water (90:10) as the mobile phase at a flow rate of 1.0 mL·min^−1^. Chromatography was performed at 40 °C and detection at 245 nm.

### 2.6. Statistical Analyses

The statistical significance of any differences between treatments was subjected to one-way analysis of variance (ANOVA). Differences with *p* < 0.05 were considered to be statistically significant. Data analyses were performed using SPSS software (SPSS Inc., Chicago, IL, USA).

## 3. Results and Discussion

### 3.1. Numbers of Cultivable Endophytic Bacteria

**Figure 1:**
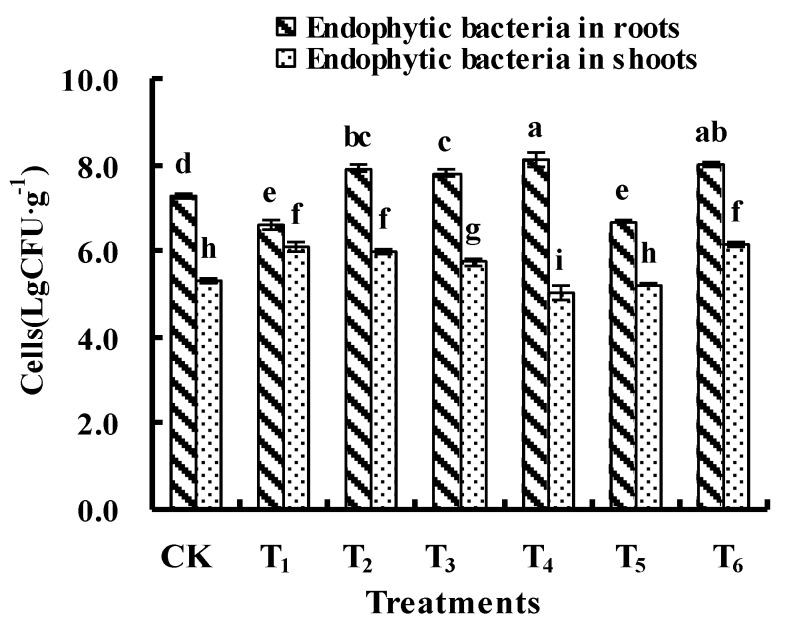
Effects of polycyclic aromatic hydrocarbons (PAHs) on the numbers of cultivable endophytic bacteria in the seedlings. Same lowercase letters indicate a lack of statistically significant difference between treatments (*p* < 0.05).

The abundance analysis indicated that exposure of ryegrass to PAHs modified the numbers of endophytic bacteria in seedlings (Figure 1). After the exposure to PAHs, increases in CFU of endophytic bacteria isolated from roots and shoots of seedlings were measured in the treatments with high concentrations of PAHs (T_2_, T_3_, and T_6_), suggesting that increasing contents of PAHs in the plant tissues would be beneficial for the growth of those endophytes that could utilize PAHs as carbon sources. On the other hand, the CFU of endophytic bacteria decreased in several treatments. The CFU of endophytic bacteria in roots increased maximally by 11.7% in group T_4_ (8.1 log CFU·g^−1^) and decreased by 9.0% in group T_1_ (6.6 log CFU·g^−1^); the CFU of endophytic bacteria in shoots increased by 14.5% in group T_1_ (6.1 log CFU·g^−1^) and decreased by 5.1% in group T_4_ (5.1 log CFU·g^−1^), which attributed to the growth inhibition of some endophytic bacterial strains by the specific concentrations of PHE (56–58 mg·kg^−1^) and PYR (~185 mg·kg^−1^) in seedlings (Table 2).

### 3.2. Diversity of Cultivable Endophytic Bacteria

**Figure 2:**
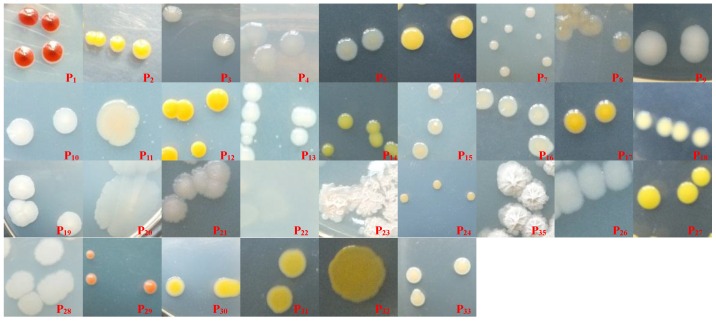
Photographs of colonies of 33 endophytic bacterial strains on Luria-Bertani medium plate.

A total of 33 endophytic bacterial strains were isolated from the seedlings in all treatments, which represented respectively 15 different genera and eight different classes (Figure 2, Table 3). After exposure to PAHs, dominant bacteria were shifted in both, roots and in shoots: nine endophytic bacterial strains, including seven *γ-Proteobacteria* strains (P_3_, P_4_, P_5_, P_8_, P_10_, P_14_, P_15_), one *Sphingobacteria* strain (P_6_), and one *α-Proteobacteria* strain (P_13_), were only isolated from the seedlings grown in solutions without PAHs; 18 endophytic bacterial strains, including 10 *γ-Proteobacteria* strains (P_16_, P_18_, P_20_, P_21_, P_24_, P_26_, P_30_, P_31_, P_32_, P_33_), four *Fusobacteria* strains (P_19_, P_23_, P_25_, P_28_), two *Sphingobacteria* strains (P_17_, P_29_), one *β-Proteobacteria* strain (P_22_), and one *Arthrobacter* strain (P_27_), were only isolated from the seedlings grown in solutions with PAHs; six endophytic bacterial strains, including three *γ-Proteobacteria* strains (P_1_, P_2_, P_9_), one *α-Proteobacteria* strain (P_7_), one *Bacilli* strain (P_11_), and one *Flavobacteria* strain (P_12_), were isolated from the seedlings grown in solutions with or without PAHs.

The bacterial strains from the genera *Bacillus*, *Pantoea*, *Pseudomonas*, *Arthrobacter*, *Pedobacter*, and *Delftia*, which were enriched only in plants exposed to PAHs, might have the capacities to specifically use PAHs as the sole carbon source. As described in previous research, strains ascribed to *Bacillus* sp. have been reported to be the efficient degraders of PAHs [13,14]. Also *Pseudomonas* sp. WJ6, a producer of rhamnolipids which act as biosurfactants, has been reported to be an excellent degrader of PAHs [15]. The presence or increase of *Pedobacter* sp. and *Flavobacteria* sp., which belong to *Sphingobacteria* and *Flavobacteria*, might be necessary for degradation of PAHs too [16]. The accumulation of PAHs in plant tissues would provide a beneficial environment for these bacterial strains while competing with other endophytic bacterial strains.

Diversity indices, including *H* (Shannon-Wiener) and *J* (Evenness) were used to assess the complexity of endophytic bacterial communities. Compared to the control, fewer bacterial strains and genera were isolated from the roots of seedlings exposed to PAHs, whereas more bacterial strains and genera were isolated from the shoots of seedlings exposed to PAHs, which might be explained by the fact that shoots were not directly exposed to PAHs, unlike the roots (Table 3). The highest biodiversity (*H* = 1.898) was found in the T_1_ group after exposure to a low PHE concentration (100 μg·L^−1^) and the poorest biodiversity (*H* = 0.610) in the T_6_ group after exposure to the mixture of high concentrations of PHE (800 μg·L^−1^) and PYR (500 μg·L^−1^), where the least types of bacteria have been found. Meanwhile, the evenness index increased from 0.603 (CK) to 0.913 (T_1_) then decreased to 0.135 (T_6_; Table 4), pointing out that the diversity and evenness can be increased by exposure to low concentrations of PHE and decreased by exposure to high concentrations of the mixed PAHs. The decreasing biodiversity caused by the mixed PAHs is in agreement with the report by Benedek et al. [16], where the biodiversity of bacterial species in soil was affected by the presence of pollutants and negatively correlated with the concentrations of PAHs.

The results of this study suggest that PHE and PYR had different influence on the endophytic bacterial diversity. After exposure of seedlings to PHE, increase of endophytic bacterial diversity occurred in roots and shoots, whereas after exposure of seedlings to PYR, the increase of the endophytic bacterial diversity only occurred in shoots. Moreover, the mixture of PHE and PYR at high concentrations (T_6_) decreased the diversity of endophytic bacterial community, although it increased the CFU of endophytic bacteria inside plants exposed to PAHs.

These findings might be ascribed to the fact that the lack of endophytic bacterial strains, which are subjected to the toxicity of mixed PHE and PYR, grew well inside plants exposed to the mixture of PHE and PYR (Table 4). Grown in the solutions with the mixed PAHs, seedlings provided beneficial environment for those bacteria, who could stand toxicity of the mixed PHE and PYR, what has resulted in an increased abundance and decreased diversity. These findings are in agreement with previous studies where it has been reported that microbial communities affected by pollution with hydrocarbons tend to be less diverse, depending on the complexity of the composition of pollutants and the time of exposure [17,18].

### 3.3. Dominant Endophytic Bacteria and Their Abilities to Degrade PAHs

A remarkable change in the endophytic bacterial community structure occurred based on the shift of dominant genera. In the control, the dominant bacteria in roots were P_3_ and P_4_ from the genus *Enterobacter* sp., and the dominant bacteria in shoots were P_12_ from the genus *Chryseobacterium* sp. and P_13_ from the genus *Agrobacterium* sp., while these endophytic bacterial strans were not from the dominant genera in the seedlings exposed to PAHs (Table 4). A high biodegradative potential of the dominant endophytic bacterial community was detected in both roots and shoots of the seedlings treated with PAHs. Similar results have been reported by Phillips et al. (2008) that the dominant endophytic bacterial communities inside the plants, which were growing in weathered-hydrocarbon contaminated soil, were associated with increased PAH degradation potential and activity [19]. Among these dominant genera, strain P_33_ from the genus *Stenotrophomonas* sp., which has been reported to degrade a range of PAHs [20], was the dominant endophyte in roots and shoots of the seedlings from the groups T_1_ and T_2_ in this study. For the seedlings grown in a solution with PYR, strain P_1_ (*Serratia* sp.), which degraded 49.2% of PYR within 7 d, became the dominant species in roots. Strain P_7_ ascribed to *Bacillus* genus, already found in contaminated soil with PAH-RHD_α_ coding genes [14], became the dominant genus in shoots from the seedlings treated with PYR.

Making a comparison of biodegradative potential of the dominant endophytic bacteria between the seedlings exposed to PHE and PYR, a high biodegradative potential of PHE was detected in both roots and shoots of the seedlings treated with PHE (more than 99% PHE was degraded within 7 d), but high biodegradative potential of PYR was not detected in the seedlings treated with PYR. This might be explained by the fact that a molecule of PYR has four benzene rings, which are recalcitrant to microbial degradation.

Apart from the differences in the composition of bacterial communities between non-exposed and exposed plants, the increase in the cells of endophytic bacteria following the addition of PAHs was observed as well. This might be due to a possible capacity of the endophytes to utilize PAHs as carbon sources, causing plants containing PAHs to be able to support a larger and adapted endophytic community in comparison to non-exposed plants. According to the increasing biodegradative potential of PAHs, pre-exposure of ryegrass plants and its associated endophytic bacteria to PAHs is suggested to be utilized to enhance the efficiency of phytoremediation of soils contaminated with PAHs.

## 4. Conclusions

PAHs modify the composition and structure of the endophytic bacterial community in ryegrass. Contamination with PAHs would be beneficial for several genera which inhabit the interior parts of plants and can potentially utilize PAHs as carbon sources. However, considering the entire endophytic bacterial community, exposure to the mixture of PAHs (PHE and PYR) may decrease its biodiversity. Most of dominant genera, such as *Bacillus* sp., *Pantoea* sp., *Pseudomonas* sp., *Arthrobacter* sp., *Pedobacter* sp., and *Delftia* sp., where only isolated from the seedlings exposed to PAHs, and dominant genera were shifted. Most of the dominant bacteria isolated from seedlings treated with PHE had higher capacities to degrade PHE (more than 99% within 7 d) than those isolated from the control. This study provides insights into the diversities and characteristics of endophytic bacterial populations in plants under PAH contamination, which might be beneficial for improving the effectiveness of remediation by plants associated with diverse endophytes.

## Figures and Tables

**Table 1:** The concentrations of PAHs in the Hoagland solution.

Group	CK	T_1_	T_2_	T_3_	T_4_	T_5_	T_6_
Phenanthrene (PHE) (μg·L^−1^)	0	100	800	0	0	100	800
Pyrene (PYR) (μg·L^−1^)	0	0	0	20	500	20	500

**Table 2:** The concentrations of PAHs in the plant tissues (mg·kg^−1^).

Group	Concentration of PAHs in the Roots		Concentration of PAHs in the Shoots	
	PHE	PYR	PHE	PYR
**CK**	5.10 ± 1.55 c	n.d.	3.38 ± 0.72 c	n.d.
**T_1_**	56.95 ± 4.98 b	n.d.	19.33 ± 0.76 bc	n.d.
**T_2_**	346.49 ± 38.18 a	n.d.	144.68 ± 14.53 a	n.d.
**T_3_**	32.14 ± 4.79 bc	31.29 ± 3.04 c	39.55 ± 10.79 b	n.d.
**T_4_**	28.10 ± 6.04 bc	812.44 ± 316.65 a	22.35 ± 1.76 bc	184.72 ± 35.34 a
**T_5_**	58.19 ± 8.55 b	30.93 ± 4.07 c	32.19 ± 9.61 b	4.37 ± 1.65 b
**T_6_**	353.45 ± 27.00 a	452.35 ± 39.66 b	152.70 ± 27.54 a	205.20 ± 101.59 a

Notes: Values are the means of three replicates ± standard error of the means. n.d. means not detected. Different lowercase letters (a, b, c, etc.) indicate statistically significant difference within the same columm (*p* < 0.05).

**Table 3:** Cultivable endophytic bacterial strains isolated from the seedlings from all groups.

Class	Genus	Bacterial Strain	Isolation from the Roots							Isolation from the Shoots						
			CK	T_1_	T_2_	T_3_	T_4_	T_5_	T_6_	CK	T_1_	T_2_	T_3_	T_4_	T_5_	T_6_
*γ-Proteobacteria*	*Enterobacter*	P_3_	+													
		P_4_	+													
		P_10_	+													
		P_14_								+						
		P_18_			+				+							
		P_20_				+						+	+			+
		P_26_			+											
	*Stenotrophomonas*	P_5_	+													
		P_8_	+													
		P_15_								+						
		P_16_			+				+							
		P_33_		+	+	+					+	+	+	+	+	
	*Pantoea*	P_30_			+				+							
		P_31_		+		+	+	+			+		+	+	+	
		P_32_			+											
	*Pseudomonas*	P_21_			+		+		+		+			+		
		P_24_				+								+		
	*Serratia*	P_1_	+		+	+	+		+		+			+		
		P_2_	+	+			+	+					+			
	*Klebsiella*	P_9_	+	+			+	+			+		+		+	
*Fusobacteria*	*Bacillus*	P_19_		+			+				+	+	+	+		+
		P_23_									+	+	+			
		P_25_				+	+	+			+		+	+		
		P_28_		+									+			
*Sphingobacteria*	*Sphingobacterium*	P_6_	+							+						
		P_17_										+				+
	*Pedobacter*	P_29_		+			+	+					+		+	
α*-Proteobacteria*	*Rhizobium*	P_7_	+	+			+	+		+		+	+	+	+	+
	*Agrobacterium*	P_13_								+						
β*-Proteobacteria*	*Delftia*	P_22_													+	
*Bacilli*	*Solibacillus*	P_11_	+		+				+							+
*Flavobacteria*	*Chryseobacterium*	P_12_					+			+	+	+	+			+
*Actinobacteria*	*Arthrobacter*	P_27_			+											
Sums of bacterial strains			11	8	10	6	10	6	6	6	9	7	12	8	6	6
Sums of genera			7	7	7	6	8	6	6	5	7	6	9	6	6	6
Sums of classes			4	4	3	2	4	4	2	4	3	5	8	3	4	6

Note: “+” means the bacteria could be isolated from the tissues of ryegrass.

**Table 4:** Effect of PAHs on populations of endophytic bacteria inside seedlings.

Plant Tissue	Group	*H*	*J*	Dominant		Number	Degradation Rate (%)	
				Bacterial Strain	Genus	Log CFU·g^−1^ (*pi*)	PHE	PYR
Roots	CK	1.447	0.603	P_4_	*Enterobacter* sp.	6.95 (45.9%)	73.4 ± 24.4	68.5 ± 8.8
				P_3_	*Enterobacter* sp.	6.78 (30.7%)	71.6 ± 31.8	25.2 ± 4.2
	T_1_	1.898	0.913	P_33_	*Stenotrophomonas* sp.	6.02 (23.5%)	99.0 ± 0.1	17.2 ± 5.9
				P_7_	*Rhizobium* sp.	5.97 (21.2%)	12.8 ± 5.4	8.7 ± 1.9
	T_2_	1.469	0.638	P_30_	*Pantoea* sp.	7.56 (41.0%)	99.9 ± 0.1	n.d.
				P_33_	*Stenotrophomonas* sp.	7.47 (33.7%)	99.0 ± 0.1	17.2 ± 5.9
	T_3_	1.370	0.764	P_1_	*Serratia* sp.	7.35 (32.0%)	97.3 ± 1.5	49.2 ± 8.2
				P_25_	*Bacillus* sp.	7.33 (31.0%)	99.8 ± 0.2	16.9 ± 9.5
	T_4_	1.039	0.451	P_1_	*Serratia* sp.	7.92 (57.1%)	97.3 ± 1.5	49.2 ± 8.2
				P_9_	*Klebsiella* sp.	7.64 (30.5%)	67.8 ± 10.5	9.2 ± 11.6
	T_5_	1.578	0.881	P_25_	*Bacillus* sp.	6.24 (35.3%)	99.8 ± 0.2	16.9 ± 9.5
				P_9_	*Klebsiella* sp.	6.17 (29.5%)	67.8 ± 10.5	9.2 ± 11.6
	T_6_	0.610	0.340	P_16_	*Stenotrophomonas* sp.	7.97 (84.6%)	68.2 ± 11.5	n.d.
				P_30_	*Pantoea* sp.	6.94 (7.9%)	99.9 ± 0.1	n.d.
Shoots	CK	1.534	0.856	P_12_	*Chryseobacterium* sp.	4.92 (38.6%)	92.5 ± 6.4	n.d.
				P_13_	*Agrobacterium*	4.65 (20.6%)	28.6 ± 2.9	46.2 ± 24.5
	T_1_	2.015	0.917	P_33_	*Stenotrophomonas* sp.	5.53 (25.8%)	99.0 ± 0.1	17.2 ± 5.9
				P_19_	*Bacillus* sp.	5.34 (16.7%)	35.3 ± 29.0	22.8 ± 15.4
	T_2_	1.768	0.908	P_7_	*Rhizobium* sp.	5.51 (29.9%)	12.8 ± 5.4	8.7 ± 1.9
				P_33_	*Stenotrophomonas* sp.	5.44 (25.4%)	99.0 ± 0.1	17.2 ± 5.9
	T_3_	2.116	0.852	P_20_	*Enterobacter* sp.	5.11 (21.6%)	14.6 ± 18.9	n.d.
				P_19_	*Bacillus* sp.	5.10 (21.3%)	35.3 ± 29.0	22.8 ± 15.4
	T_4_	2.033	0.978	P_33_	*Stenotrophomonas* sp.	4.36 (19.1%)	99.0 ± 0.1	17.2 ± 5.9
				P_25_	*Bacillus* sp.	4.33 (17.7%)	99.8 ± 0.2	16.9 ± 9.5
	T_5_	1.412	0.788	P_7_	*Rhizobium* sp.	4.86 (41.5%)	12.8 ± 5.4	8.7 ± 1.9
				P_9_	*Klebsiella* sp.	4.72 (29.5%)	67.8 ± 10.5	9.2 ± 11.6
	T_6_	1.159	0.647	P_19_	*Rhizobium* sp.	6.00 (66.4%)	35.3 ± 29.0	22.8 ± 15.4
				P_7_	*Bacillus* sp.	5.16 (9.4%)	12.8 ± 5.4	8.7 ± 1.9

Note: n.d. means the biodegradation could be ignored.
